# Tunnel Anastomosis vs Double-Tract Jejunal Interposition Reconstruction After Proximal Gastrectomy: Protocol for a Multicenter Prospective Randomized Controlled Trial

**DOI:** 10.2196/82712

**Published:** 2026-03-27

**Authors:** Qingyu Xie, Rui Peng, Chao Yue, Wei Wei, Lingli Huang, Xiaoxiao Wang, Haitian Wang, Liang Chen, Rongmin Gu, Huanqiu Chen, Xuezhi Ming, Xu Wen, Weiguo Xu, Guangli Sun, Hao Fan, Zhe Wang, Longhao Yang, Xiaohua Zhou, Xiaoyu Wu, Jin Zhou, Meng Wang, Hao Xu, Gang Li

**Affiliations:** 1 The Affiliated Cancer Hospital of Nanjing Medical University & Jiangsu Cancer Hospital & Jiangsu Institute of Cancer Research, No. 42, Baiziting, Nanjing, 210009, China, 86 13776689750; 2 The Affiliated Hospital of Nanjing University of Chinese Medicine, Nanjing, China; 3 Jiangsu University Affiliated Gaochun Hospital, Nanjing, China; 4 The First Affiliated Hospital of Soochow University, Suzhou, China; 5 Nanjing University Medical School Affiliated Drum Tower Hospital, Nanjing, China; 6 The First Affiliated Hospital of Nanjing Medical University, Nanjing, China

**Keywords:** gastric cancer, proximal gastrectomy, tunnel anastomosis, double-tract jejunal interposition reconstruction, antireflux

## Abstract

**Background:**

Tunnel anastomosis is a novel anastomotic technique for digestive tract reconstruction following proximal gastrectomy. A previous retrospective study by our team demonstrated its favorable antireflux effect; therefore, we hypothesize that tunnel anastomosis is noninferior to double-tract jejunal interposition reconstruction in preventing postoperative reflux esophagitis, and we will conduct this prospective study to further validate this assumption.

**Objective:**

In this study, we will prospectively compare tunnel anastomosis with the currently more prevalent double-tract jejunal interposition reconstruction technique to further validate its safety and efficacy.

**Methods:**

This is a multicenter prospective randomized controlled study that will enroll 240 patients who will undergo proximal gastrectomy. The study will be divided into 2 groups: the tunnel anastomosis group and the double-tract jejunal interposition reconstruction group, with 120 patients in each group. Patients will undergo clinical assessments and complete questionnaires preoperatively, as well as at the 3rd, 6th, and 12th months postoperatively. The primary end point is the incidence of reflux esophagitis within 1 year. The secondary end points include perioperative safety, postoperative quality of life, and postoperative nutritional status.

**Results:**

Recruitment of patients commenced in March 2022 and is scheduled to conclude in February 2027. The follow-up for all enrolled patients will be completed by February 2028.

**Conclusions:**

To our knowledge, this is the first prospective study on this technique, aiming to provide novel insights into the methods of digestive reconstruction following proximal gastrectomy.

## Introduction

Gastric cancer is a type of cancer with high morbidity and mortality in China, and the incidence of proximal gastric cancer is currently increasing [1-3]. Surgical treatment for proximal gastric cancer involves either total gastrectomy or proximal gastrectomy. Research has shown that proximal gastrectomy has the same therapeutic potential and efficacy as total gastrectomy, with advantages in terms of postoperative weight loss, dumping syndrome, anemia, and nutritional supplementation [4-10]. However, the issue of reflux esophagitis following proximal gastrectomy remains a significant challenge for surgeons [11]. Numerous studies have been conducted on antireflux techniques for digestive reconstruction, including double-tract jejunal interposition reconstruction and the double-flap technique (Kamikawa anastomosis), which have exhibited promising antireflux effects. Nevertheless, there is still no consensus on which reconstruction method should be considered the standard [12-17].

Tunnel anastomosis is a novel technique for digestive tract reconstruction following proximal gastrectomy. Owing to its preservation of an intact muscular flap, it ensures superior blood supply to the anastomotic site. A previous study by our team demonstrated that the antireflux effects of this technique are favorable [18]. Double-tract jejunal interposition reconstruction is currently the mainstream method for digestive tract reconstruction following proximal gastrectomy and is widely recognized by most experts [19]. The incidence of postoperative reflux esophagitis following this procedure is approximately 10%. Therefore, we selected this technique as the control group to further validate the effectiveness of TA.

We hypothesize that tunnel anastomosis is noninferior to double-tract jejunal interposition reconstruction in preventing postoperative reflux esophagitis. The objective of this study is to further validate the surgical safety and antireflux effect of tunnel anastomosis and assess the efficacy and ability of this technique to improve the quality of life of patients following proximal gastrectomy. It is hoped that this research will contribute to enhancing the theoretical and clinical practice foundations for refining surgical treatment strategies for upper gastric cancer.

## Methods

### Ethical Considerations

This study has been approved by the ethics committee of the Affiliated Cancer Hospital of Nanjing Medical University (2021-091-01). All patients will be fully informed of the precautions by a professional physician and provided with a written informed consent form before participation. All research information will be kept strictly at the study site and these data will be anonymized. This information will not be published outside the research without the consent of the patients.

### Study Design and Participants

**Figure 1.**
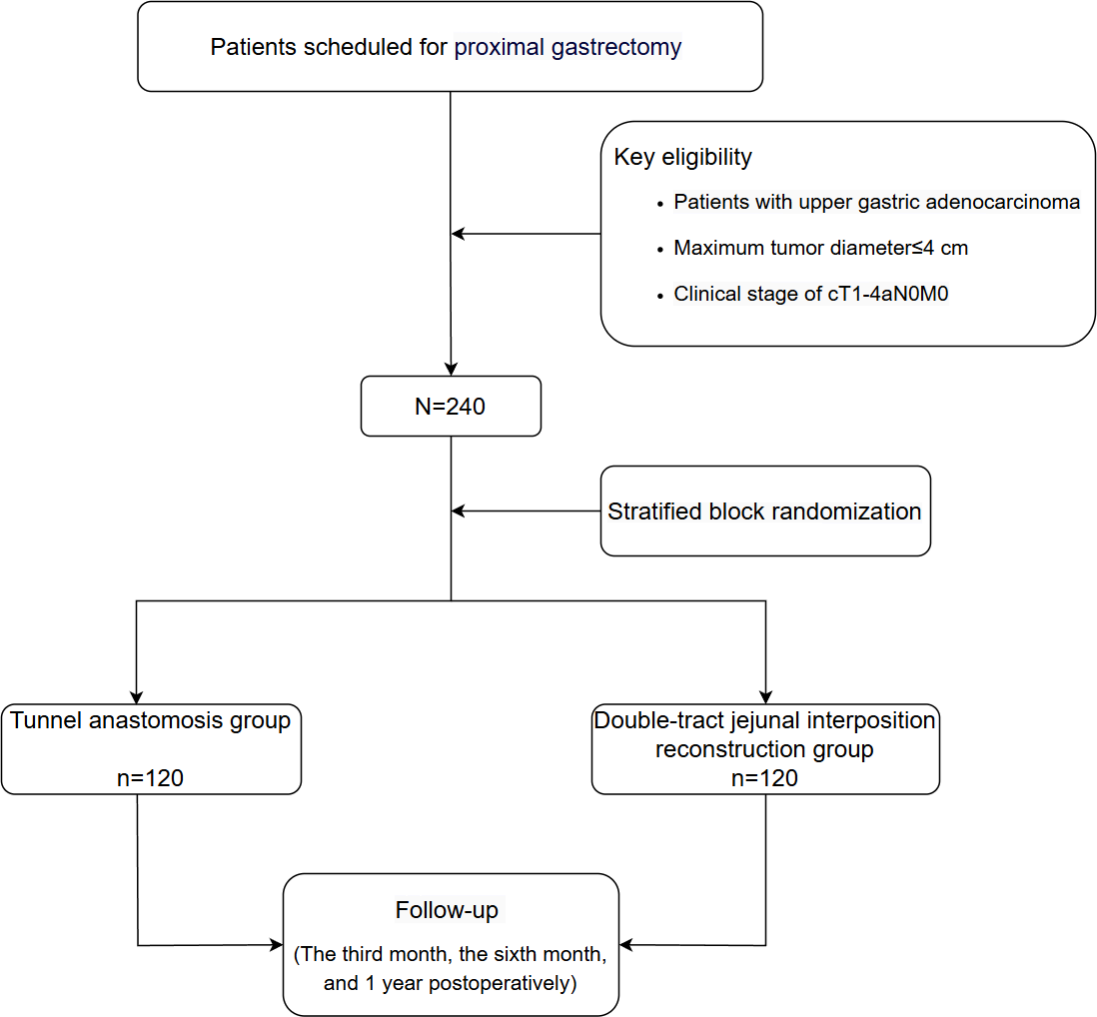
Flow diagram for the study.

This multicenter prospective study will be conducted at the Affiliated Cancer Hospital of Nanjing Medical University, First Affiliated Hospital of Nanjing Medical University, Affiliated Drum Tower Hospital of Nanjing University Medical School, First Affiliated Hospital of Soochow University, Affiliated Hospital of Nanjing University of Chinese Medicine, and Affiliated Gaochun Hospital of Jiangsu University. Figure 1 illustrates the flow of the study.

The inclusion criteria for this study are as follows: (1) age range of 18 to 80 years, with no sex preference; (2) histopathological confirmation of adenocarcinoma via endoscopic biopsy; (3) tumor located in the upper third of the stomach without esophageal involvement, largest diameter of ≤4 cm, and preoperative clinical stage cT1-4aN0M0; (4) Eastern Cooperative Oncology Group Performance Status score of 0 to 1; (5) no surgical contraindications identified through comprehensive preoperative evaluations; and (6) voluntary and informed consent signed by the patient or their legal representative. The exclusion criteria are as follows: (1) pregnant or lactating women; (2) presence of severe psychiatric disorders; (3) intraoperative findings indicating tumor invasion into the esophagus or unsuitability for proximal gastrectomy as determined by the primary surgeon; (4) preoperative or intraoperative discovery of distant organ metastasis or extensive peritoneal implantation metastasis; (5) presence of concurrent or metachronous malignancies, including other organ tumors; (6) incomplete radical surgery, including patients who underwent palliative tumor resection; (7) a history of gastrointestinal surgery; (8) a history of neoadjuvant radiotherapy or chemotherapy; (9) serious concomitant diseases that may make the survival period <5 years; and (10) cases considered unsuitable by the investigator.

### Postrecruitment Withdrawals and Exclusions

Patients can withdraw from this study at any time. For patients who withdraw, the information collected before withdrawal will be used for the final analysis unless they request that their information be deleted.

### Preoperative Clinical Staging

Before surgery, all patients will be required to undergo an endoscopic examination with biopsy, endoscopic ultrasonography, a plain scan and a contrast-enhanced scan of the whole abdomen, and an upper gastrointestinal series. This comprehensive assessment aims to evaluate the gastric cancer lesion and abdominal lymph nodes thoroughly, with the objective of obtaining an accurate preoperative clinical diagnosis and tumor staging.

### Randomization

Patients can only be randomized into different groups once they have signed the informed consent form, completed baseline assessments, and been confirmed as fully meeting the inclusion criteria. Eligible patients will be randomly assigned to 1 of the following 2 groups at a 1:1 ratio: the tunnel anastomosis group or the double-tract jejunal interposition reconstruction group. The randomization process will use a stratified block randomization method, with disease stage (categorized as early stage or advanced stage) serving as the stratification factor. Patient group assignment will be determined based on random numbers generated by the R software program (version 4.0.2; R Foundation for Statistical Computing).

### Surgical Procedure

#### Overview

Both groups will undergo proximal gastrectomy with radical lymph node dissection (open, laparoscopic, or robot-assisted). cT1N0 patients will undergo D1+ lymph node dissection (1, 2, 3a, 4sa, 4sb, 7, 8a, 9, and 11p), whereas the remaining patients will undergo D2 lymph node dissection (D1+ and 11d). The surgeon will be required to hold the qualification of a chief physician and strictly adhere to the protocol procedures for reconstruction. All surgical procedures will be video or photo recorded.

#### Tunnel Anastomosis Technique

**Figure 2.**
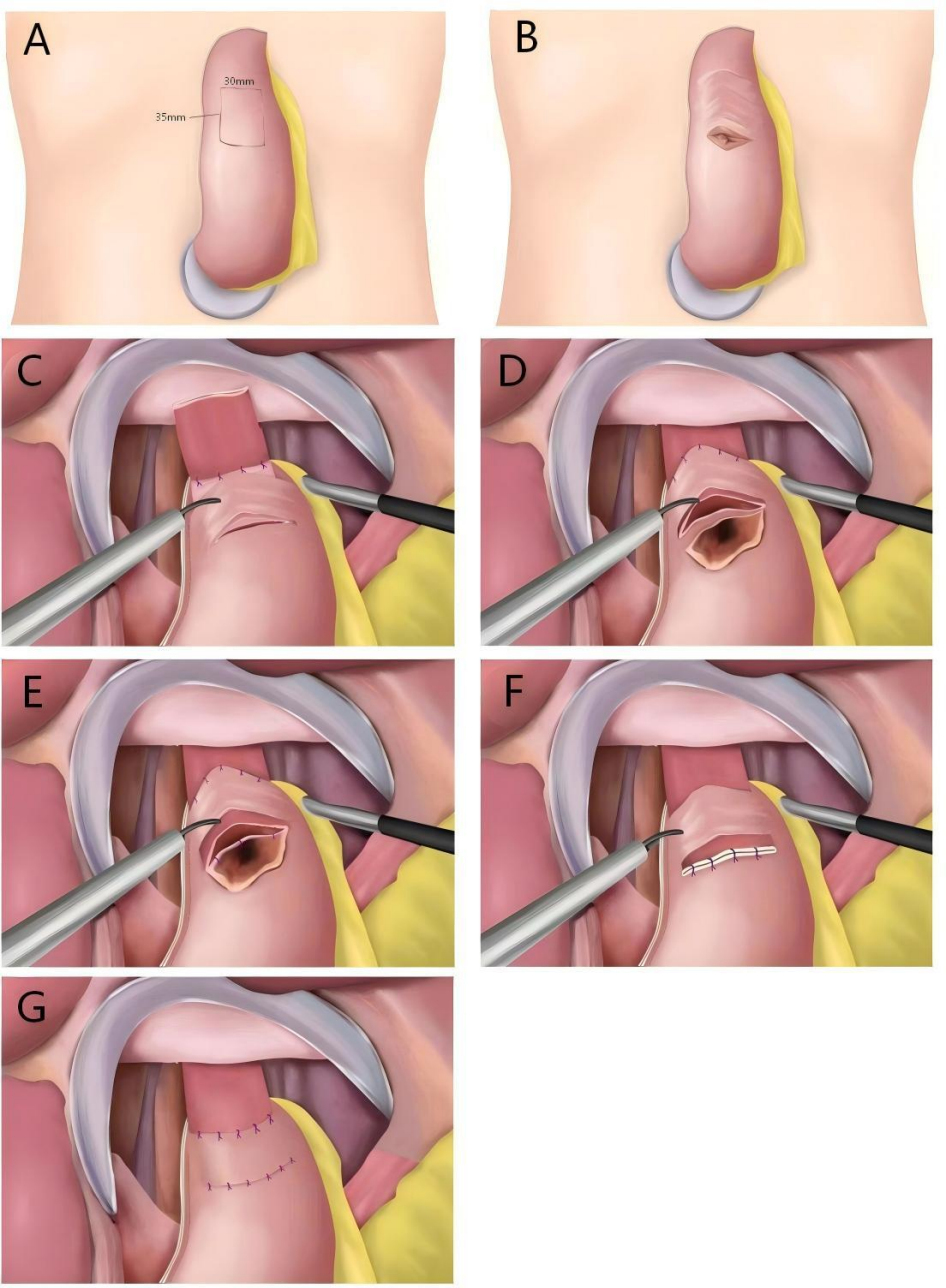
Surgical steps for tunnel anastomosis.

First, a linear cutting stapler will be used to transect the esophagus and create a gastric tube. Second, a 3-cm transverse incision will be made in the anterior wall of the remnant stomach, approximately 3 to 4 cm from the upper edge between the greater and lesser curvatures, reaching but not incising the muscular layer. Third, another parallel incision of equal length 3.5 cm distal to the first incision will be made. Fourth, between these 2 incisions, the connective tissue between the submucosa and muscular layers will be dissected via an electrosurgical knife, creating a tunnel flap of approximately 3 × 3.5 cm. Fifth, the posterior wall of the esophagus, located 5 cm from the residual stump, will be sutured with 4 stitches to the gastric wall at the upper edge of the seromuscular flap. Sixth, the esophageal stump will be pulled through the tunnel, and the anterior wall of the esophagus will be sutured with 4 to 5 stitches to the upper edge of the gastric seromuscular flap. Seventh, the submucosa and mucosal layers of the stomach will be incised at the lower incision of the tunnel to prepare for anastomosis with a caliber of 3 cm. Eighth, the esophageal stump will be opened using an ultrasonic knife, and the posterior wall of the esophagus will be sutured to the gastric mucosa and submucosa. Ninth, the anterior wall of the esophagus will be sutured to the full layer of the stomach. Finally, the lower edge of the seromuscular flap and the seromuscular layer of the remnant stomach will be sutured (Figure 2) [18].

#### Double-Tract Jejunal Interposition Reconstruction Technique

First, mesenteric vessels will be ligated 15 to 20 cm from the Treitz ligament. Second, the distal jejunum will be pulled anterior to the transverse colon and anastomosed with the esophagus. Third, approximately 10 to 15 cm below the esophagojejunal anastomosis, the distal jejunum will be anastomosed with the anterior wall of the remnant stomach. A 6-cm linear stapler will be used to create an anastomosis with a size of 4 cm. Subsequently, at 30 to 35 cm from the gastrojejunal anastomosis, a second anastomosis will be performed between the proximal and distal jejunum.

### Follow-Up

Follow-up will be conducted by dedicated follow-up staff. All patients will undergo follow-up visits at 3 months, 6 months, and 1 year postoperatively. At these visits, hematological examinations and postoperative quality of life assessments (using the European Organisation for Research and Treatment of Cancer Quality of Life Questionnaire Core 30 [EORTC QLQ-C30] and 36-Item Short Form Health Survey) will be performed. Nutritional status will be assessed based on hematological test results, changes in body weight, and the prognostic nutritional index (PNI). In addition, reflux symptoms will be assessed via the Visick score. At the 1-year follow-up, gastroscopy will be performed to assess reflux esophagitis according to the Los Angeles (LA) classification.

### Assessment of Outcomes

#### Primary End Points

The primary end point is the incidence of reflux esophagitis, which will be determined via gastroscopy results 1 year postoperatively. To determine the severity of reflux esophagitis, the modified LA classification system for reflux esophagitis will be used, which is considered a reliable method for categorizing reflux esophagitis. The modified LA classification criteria are as follows [20]:

- Grade N—normal mucosa
- Grade M—minimal changes to the mucosa, such as erythema and/or whitish turbidity
- Grade A—nonconfluent mucosal breaks of <5 mm in length
- Grade B—nonconfluent mucosal breaks of >5 mm in length
- Grade C—confluent mucosal breaks <75% circumferential
- Grade D—confluent mucosal breaks >75% circumferential

#### Secondary End Points

The secondary study end points include perioperative safety, postoperative nutritional status, and postoperative quality of life.

Perioperative safety primarily encompasses the duration of surgery, amount of intraoperative blood loss, and postoperative complication status. Nutritional status and quality of life following surgery will be assessed at the 3rd, 6th, and 12th postoperative months. Postoperative nutritional status will be determined based on changes in patient weight, hemoglobin levels, total protein levels, albumin levels, total lymphocyte count, and PNI. Additionally, patients’ quality of life will be evaluated via the EORTC QLQ-C30 and daily food intake frequency. The Visick score will be used for assessing patients’ symptoms and quality of life [21]:

- Grade I—patients are asymptomatic or have only mild symptoms that do not significantly affect their quality of life. Surgical outcomes are considered excellent.
- Grade II—patients experience mild symptoms that have minimal impact on their daily activities. Overall, patients are satisfied with the surgical outcome.
- Grade III—patients have moderate to severe symptoms that affect their daily lives. These symptoms may require medical treatment or lifestyle modifications. The surgical outcome is considered less favorable.
- Grade IV—patients have severe symptoms that significantly impact their quality of life. The surgical procedure has failed to achieve the desired outcome. Further surgical intervention or other treatments may be necessary.

### Power and Sample Size

This study is designed as a prospective randomized controlled trial with a noninferiority objective. It comprises 2 groups: the experimental group (tunnel anastomosis group) and the control group (double-tract jejunal interposition reconstruction group).

The reported incidence of reflux esophagitis for DTJIR in the literature varies widely, ranging from 1.7% to 25%. However, most contemporary studies report rates concentrated at approximately 10% [22-24]. On the basis of retrospective data from our center, the incidence of reflux esophagitis for TA is approximately 5%. Therefore, the estimated incidence of reflux esophagitis was 10% in the DTJIR group and 5% in the TA group. With a significance level set at α=.025 (one sided), a power of 1 – β of 0.80, a noninferiority margin of 0.05 (given the reported incidence of reflux esophagitis with the current surgical options, a noninferiority margin of ≤5% was deemed clinically acceptable as such a difference would not offset the significant benefits associated with the new technique), a 1:1 ratio between the experimental and control groups, and an anticipated dropout rate of 10%, the sample size was calculated via the PASS software (NCSS, LLC). The resulting sample sizes for both groups were 120 patients each.

Therefore, a total of 240 patients will be included in this study, with 120 patients in the experimental group and 120 patients in the control group.

### Data Collection

Basic patient information and clinical data will be collected before discharge, including identity card number; gender; age; contact information; date of operation; surgical approach; duration of surgery; intraoperative blood loss; pathological tumor, node, metastasis staging; number of lymph nodes dissected; number of positive lymph nodes; length of postoperative hospital stay; and postoperative complications. Each patient will be followed up on at the 3rd, 6th, and 12th months postoperatively, and the following data will be collected regarding their postoperative quality of life: daily meal frequency, Visick score, and postoperative quality of life score (using the EORTC QLQ-C30), as well as data on their postoperative nutritional status, including weight change, hemoglobin level, total protein level, albumin level, total lymphocyte count, and PNI.

The basic information and clinical data for each patient collected before discharge will be classified, stored, and entered into the database. The results of each follow-up will be meticulously recorded and entered into the database alongside the other data. All electronic data will be backed up to prevent loss.

### Statistical Analysis

Data analysis will be conducted by professional statisticians. In addition to the overall comparative analysis conducted between the TA and DTJIR groups, we will further perform subgroup analyses stratified by disease stage and surgical approach.

Intention-to-treat and per-protocol approaches will be applied for efficacy analysis. An as-treated approach will be used for safety analysis. For continuous variables, a normality test will be performed first. For those that conform to a normal distribution, the 2-tailed *t* test will be used for statistical analysis (all continuous values will be expressed as means and SDs). Those not conforming to a normal distribution will be presented as quartiles and rank means, and the Mann-Whitney *U* test will be used to calculate the *P* value to compare differences between groups. For categorical variables, the chi-square test will be used for statistical analysis. Repeated-measure data will be analyzed using a mixed-effects model. Subgroup analyses will be conducted by disease stage (early vs advanced); surgical approach (open, laparoscopic, or robot-assisted); age; macroscopic types; and tumor, node, metastasis stage. All *P* values calculated in the analysis will be 2-sided, and *P*<.05 will be considered statistically significant. If missing data constitute more than 5%, multiple imputations will be conducted. Statistical analyses will be performed via SPSS (IBM Corp), Prism (GraphPad Software), or PASS.

## Results

The first patient was enrolled in March 2022, and the recruitment of all 240 patients is expected to be completed by February 2027. Follow-up for these patients is scheduled to conclude in February 2028.

## Discussion

This study may demonstrate that tunnel anastomosis is an alternative method of digestive tract reconstruction following proximal gastrectomy that is as effective as DTJIR. Its antireflux effect may make it one of the most widely used methods of digestive tract reconstruction after proximal gastrectomy.

Proximal gastrectomy is gaining increasing acceptance among the medical community due to its advantages in postoperative nutrition [25]. However, for patients undergoing proximal gastrectomy, postoperative reflux esophagitis is a significant issue. Various anastomotic techniques have been investigated in an attempt to address this problem, yet a standard approach remains elusive [24]. In pursuit of a superior method for digestive tract reconstruction, we modified the Kamikawa anastomosis to develop tunnel anastomosis and conducted a retrospective study. The retrospective study found that tunnel anastomosis is as effective as DTJIR at preventing reflux esophagitis [15,18]. To further validate the preventive effect against reflux esophagitis of this technique, we will conduct this prospective study with the aim of providing a novel reference for digestive tract reconstruction following proximal gastrectomy. Compared with our previous study, this research has a clearly defined objective and rigorous structure. It can also reduce recall bias and control for confounding factors more effectively, yielding more reliable outcomes. Achieving strict adherence to the study protocol will also take longer.

To our knowledge, this is the first prospective study on this technique. To ensure the quality of the enrolled patients, we used stringent inclusion and exclusion criteria in this study. As this is a multicenter study, surgeons are required to possess extensive surgical experience to ensure the quality of the surgical procedures. The operative processes of patients will be documented through video and photographic records. We have also meticulously designed the subsequent follow-up and data analysis protocols. This well-considered design leads us to expect a genuine and reliable conclusion.

This study also has certain inherent limitations. It is not possible to blind patients, surgeons, radiologists, or clinical assessors in this trial. Both the physicians and patients have a clear understanding of the surgical procedure that will be performed. To minimize the bias introduced by the inability to implement blinding, the follow-up and data analysis will be conducted by different people. Meanwhile, as a prospective study, it may have the capacity to mitigate the impact of such bias on the results. The primary end point of this study is the 1-year incidence rate of reflux esophagitis. However, this fails to reflect the long-term antireflux effect. In the future, we will conduct longer-term follow-up to investigate the long-term efficacy of tunnel anastomosis. To increase the dissemination of our research, we will also present our study and its results at a wide range of academic conferences.

## Supplementary material

10.2196/82712 Checklist 1 SPIRIT checklist.

## Abbreviations

DTJIR: double-tract jejunal interposition reconstruction
EORTC QLQ-30: European Organisation for Research and Treatment of Cancer Quality of Life Questionnaire Core 30
LA: Los Angeles
PNI: prognostic nutritional index
TA: tunnel anastomosis
